# Chronic alcohol ingestion exacerbates skeletal muscle myopathy in HIV-1 transgenic rats

**DOI:** 10.1186/1742-6405-8-30

**Published:** 2011-08-16

**Authors:** Caroline R Clary, Daniel M Guidot, Margaux A Bratina, Jeffrey S Otis

**Affiliations:** 1 Department of Medicine, Emory University School of Medicine, 615 Michael Street, Atlanta, GA 30322, USA

## Abstract

**Background:**

Separately, chronic alcohol ingestion and HIV-1 infection are associated with severe skeletal muscle derangements, including atrophy and wasting, weakness, and fatigue. One prospective cohort study reported that 41% of HIV-infected patients met the criteria for alcoholism, however; few reports exist on the co-morbid effects of these two disease processes on skeletal muscle homeostasis. Thus, we analyzed the atrophic effects of chronic alcohol ingestion in HIV-1 transgenic rats and identified alterations to several catabolic and anabolic factors.

**Findings:**

Relative plantaris mass, total protein content, and fiber cross-sectional area were reduced in each experimental group compared to healthy, control-fed rats. Alcohol abuse further reduced plantaris fiber area in HIV-1 transgenic rats. Consistent with previous reports, gene levels of myostatin and its receptor activin IIB were not increased in HIV-1 transgenic rat muscle. However, myostatin and activin IIB were induced in healthy and HIV-1 transgenic rats fed alcohol for 12 weeks. Catabolic signaling factors such as TGFβ_1_, TNFα, and phospho-p38/total-p38 were increased in all groups compared to controls. There was no effect on IL-6, leukemia inhibitory factor (LIF), cardiotrophin-1 (CT-1), or ciliary neurotrophic factor (CNTF) in control-fed, transgenic rats. However, the co-morbidity of chronic alcohol abuse and HIV-1-related protein expression decreased expression of the two anabolic factors, CT-1 and CNTF.

**Conclusions:**

Consistent with previous reports, alcohol abuse accentuated skeletal muscle atrophy in an animal model of HIV/AIDS. While some catabolic pathways known to drive alcoholic or HIV-1-associated myopathies were also elevated in this co-morbid model (e.g., TGFβ_1_), consistent expression patterns were not apparent. Thus, specific alterations to signaling mechanisms such as the induction of the myostatin/activin IIB system or reductions in growth factor signaling via CT-1- and CNTF-dependent mechanisms may play larger roles in the regulation of muscle mass in alcoholic, HIV-1 models.

## Introduction and hypotheses

Multi-organ pathologies have been well-described in a host of systemic disease states. For example, chronic alcoholism and HIV/AIDS infection are each associated with liver and kidney complications, immune dysfunction, and cardio-respiratory alterations [1-5]. Unfortunately, the National Institutes of Alcohol Abuse and Alcoholism (NIAAA) confirmed that people infected with HIV-1 are more likely to abuse alcohol at some time during their lives [6,7] which would likely increase the incidence of health complications in this population. Further, these co-morbid alcoholic and HIV/AIDS pathologies may become more clinically apparent when considered in parallel with increased patient survival rates due to effective antiretroviral therapy. These co-morbid effects, which may include memory loss and brain damage [8,9], cardiomyopathy [10,11], compromised immune function [12] with increased susceptibility to opportunistic infection [13], are often severe and associated with lower perceived qualities of life compared to otherwise healthy alcoholics or HIV-1 infected patients [14]. Significant skeletal muscle derangements such as atrophy, weakness, and dysfunction are evident in both disease states [15-19]. However; few reports exist that describe the co-morbid effects of chronic alcohol ingestion and HIV-1 on skeletal muscle biology [10,20].

In simian immunodeficiency virus (SIV)-infected Rhesus macaques, chronic binge-drinking during the asymptomatic phase of the disease increased viral load and skeletal muscle expression of inflammatory cytokines while altering nutritional intake, but did not alter rates of skeletal muscle protein synthesis or degradation [20]. However, as viral load increased and SIV infection progressed to more terminal stages (SAIDS), alcohol abuse increased expression of two muscle-specific E3 ubiquitin ligases, atrogin-1 and muscle ring finger protein-1, and drove skeletal muscle proteolysis [10]. These data clearly show that binge-like alcohol abuse exacerbates HIV-associated myopathy during the progression of the disease. Here, we extend these critical observations by Molina and colleagues by using a commercially available, infection- and replication-deficient rat model of HIV-1 that develops many AIDS-like pathologies such as systemic muscle atrophy and weight loss, neurological abnormalities, and respiratory and immune dysfunctions [3,15,21-23],

Interestingly, AIDS-like pathologies can be replicated using *in vivo *and *in vitro *models without the influence of HIV-1 viral burden [3,23-26]. For example, evidence has implicated HIV-1-related proteins, such as gp120 and Tat, as mediators of injury even when target cells are not directly infected with HIV-1. Specifically, we have shown that atrophied skeletal muscles from HIV-1 transgenic rats have altered glutathione states and increased expression of atrogin-1 and TGFβ_1 _[15] - metabolic and biochemical responses we have also documented in otherwise-healthy, alcoholic muscle [16,27].

Based on this previous work, the current study was designed to identify the catabolic effects of chronic alcohol ingestion and its influence on viral-independent mechanisms of HIV-associated myopathy. Accordingly, we fed a subset of HIV-1 transgenic rats the Lieber-DeCarli liquid diet plus alcohol for 12 weeks. We hypothesized that long term alcohol abuse would exacerbate skeletal muscle atrophy, drive expression of the cell cycle regulator myostatin and its receptor activin IIB, alter expression of several catabolic and anabolic IL-6 family members, drive gene expression of TNFα, and increase p38 MAPK phosphorylation [16,28-33].

## Results and discussion

**Figure 1:**
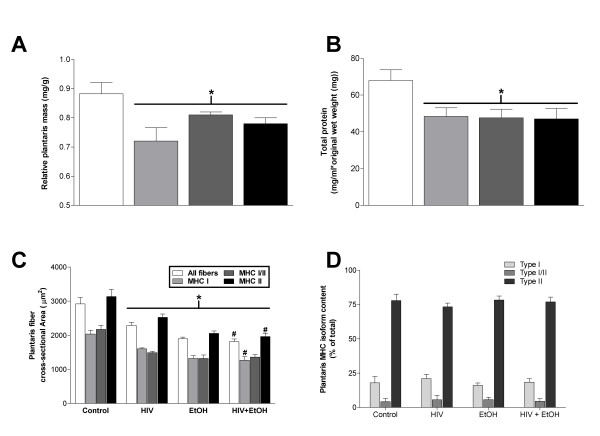
**Chronic alcohol ingestion exacerbates plantaris fiber area in HIV-1 transgenic rats**. (A) Relative plantaris mass, (B) total protein content, and (C) fiber area were reduced in each disease state. Myosin heavy chain isoform (MHC) content was unchanged in any group (D). Twelve weeks of chronic alcohol ingestion further reduced plantaris fiber area in HIV-1 transgenic rats compared to control-fed transgenic rats (C). A portion of this figure has been previously published [15]. Values are expressed as means + SEM (n = 6-7 rats/group). Significance was accepted at p ≤ 0.05. *, compared to control-fed, otherwise-healthy rat group. #, compared to control-fed, HIV-1 transgenic rat group.

**Figure 2:**
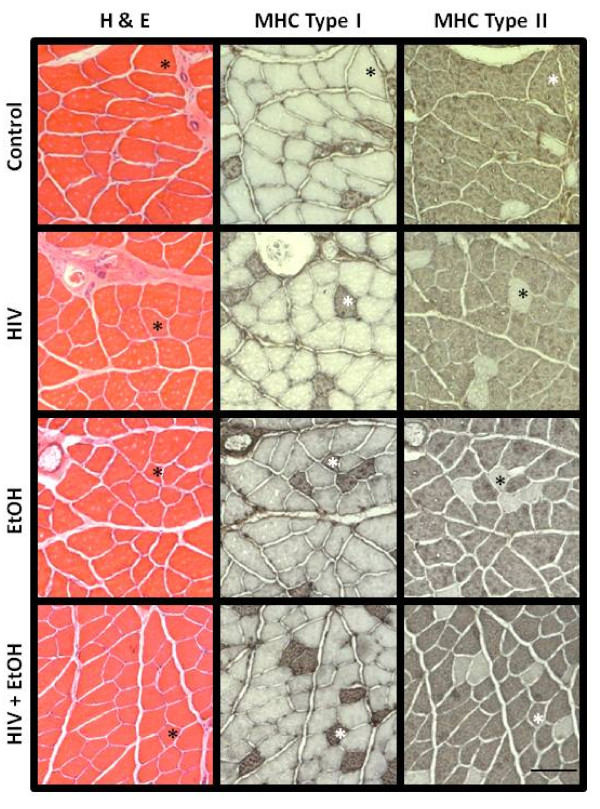
**Representative (immuno)histological staining in alcoholic plantaris muscles from HIV-1 transgenic rats**. Measurements of fiber area (quantified in Figure 1C) were made from hematoxylin and eosin (H&E-stained) sections. Serial sections were also processed for immunohistochemical detection of MHC type I and/or MHC type II (IIa, IIx, and IIb) expression. Hybrid fibers - those that stain positively for both MHC type I and any one of the three fast MHC isoforms in rat muscle - are identified by the asterisks in the HIV + EtOH panels. For convenience, asterisks have been provided within each group to track fibers in series.

We have previously described plantaris atrophy in HIV-1 transgenic rats (republished in part in Figures 1A and 1C) and now show concomitant decreases in total protein content (Figure 1B) compared to age- and gender-matched wild type controls [15]. These myopathies are readily apparent in alcoholic muscle as well (Figure 1B) and occur independent of myosin heavy chain isoform (MHC) expression (Figure 1C). Moreover, MHC isoform content was unchanged in any group (Figures 1D, 2). As expected, plantaris fiber area was further reduced in alcoholic, transgenic rats (Figure 1C). These data confirmed earlier reports from terminal stage SIV-infected (SAIDS) Rhesus macaques that alcohol ingestion exacerbates skeletal muscle wasting [10].

In both animal models of HIV/AIDS, the quantity and duration of alcohol abuse, in combination with the stage of disease progression, may have significant influence on the development of overt skeletal muscle atrophy. For example, in the early stages of SIV infection, binge-like alcohol ingestion in asymptomatic Rhesus macaques did not alter skeletal muscle protein synthesis nor degradation rates. However, alcohol did increase viral load and drive expression of tumor necrosis factor-α in skeletal muscle and may reflect the early establishment of a pro-atrophy program. If we had initiated our alcohol feeding paradigm during a similar, asymptomatic stage in the HIV-1 transgenic rat model (e.g., less than 5 months of age), we might have expected muscle adaptations that reflected the alcoholic, asymptomatic, SIV-infected Rhesus macaques. Our chronic alcohol feeding paradigm was started in HIV-1 transgenic rats that already displayed significant myopathies [15] and support the notion that alcohol abuse exacerbates HIV-1 associated skeletal muscle derangements [10]. Together, these data clearly confirm the importance of HIV-1 infected patients to avoid binge-like or chronic alcohol abuse or to aggressively seek treatment options to minimize the co-morbid impact of the diseases.

**Figure 3:**
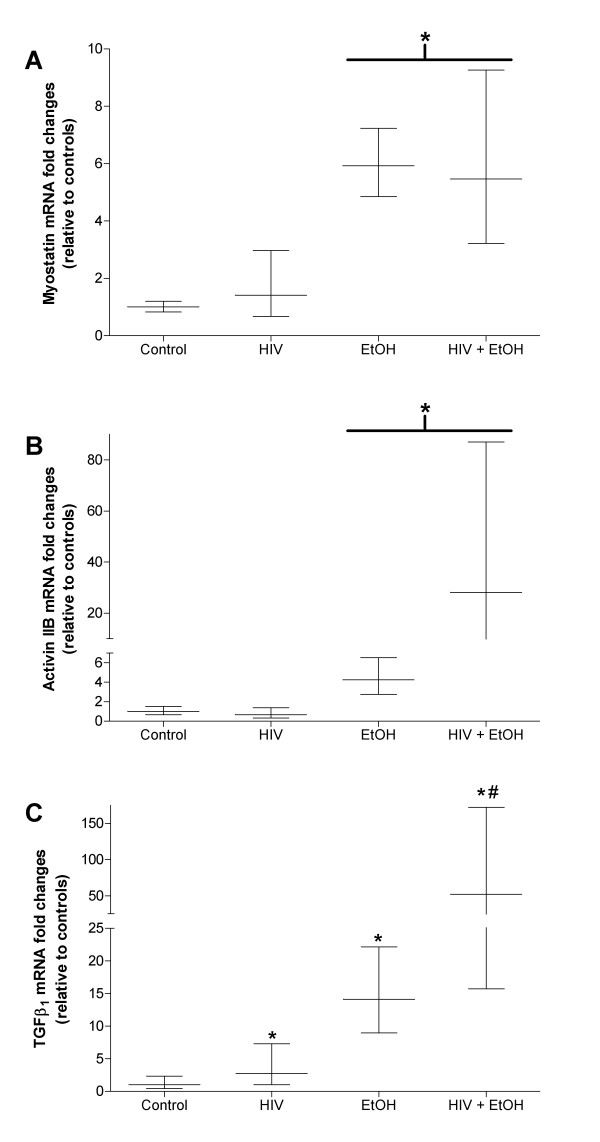
**Gene levels of TGFβ super family members are differentially regulated in diseased plantaris muscles**. Gene expression of myostatin (A) and its receptor activin IIB (B) were up-regulated in alcoholic plantaris muscles regardless of HIV-1-related protein expression. In contrast, TGFβ_1 _was up-regulated in each disease state (C). Unlike myostatin and activin IIB, TGFβ_1 _gene levels were higher in the co-morbid model compared to plantaris muscles from control-fed, HIV-1 transgenic rats. Data are represented as means ± range of potential values based on the 2^-ΔΔC^_T _method [15,16] and expressed as fold changes relative to controls (n = 6-7 rats/group). Significance was accepted at p ≤ 0.05. *, compared to control-fed, otherwise-healthy rat group. #, compared to control-fed, HIV-1 transgenic rat group.

Myostatin, a member of the TGFβ super family and implicated in various models of skeletal muscle atrophy, likely influences skeletal muscle mass via cell cycle regulation and alterations to myogenic stem cells [34-39]. We confirm earlier reports that myostatin is upregulated in alcoholic muscle [36] (Figure 3A) and show comparable alcohol-induced increases to its receptor, activin IIB (Figure 3B). Alterations to myostatin and activin IIB were also evident in muscles from alcoholic, HIV-1 transgenic rats. However, neither gene product was elevated in control-fed, transgenic rat muscles signifying that chronic alcohol ingestion may be the significant insult that drives myostatin and activin IIB expressions, not the associated pathology of HIV-1-related proteins. Similarly, myostatin mRNA expression levels were unchanged in muscles from asymptomatic or SAIDS macaques [10] and support the notion that neither viral infection nor HIV-1-related protein expression impact myostatin signaling. However, this contention is controversial as elevated serum and intramuscular levels of myostatin appeared to play a role in AIDS-associated wasting in HIV-infected men [40]. These discrepancies in myostatin expression levels may be due in part to variations between models (i.e., murine, simian, and human), disease stage and progression, or nutritional and metabolic abnormalities. More work is required to clarify the involvement of myostatin signaling in HIV-1 associated myopathies.

We also confirmed earlier reports that both alcohol abuse and HIV-1-related protein expression induce TGFβ_1 _gene expression [15,27], an effect that was further increased in the co-morbid condition (Figure 3C). Predictably, the catabolic influences of chronic alcohol ingestion and HIV-1-related protein expression impact TGFβ super family members in various degrees. Although speculative, treatment options that affect a broad range of factors in skeletal muscle (e.g., anti-oxidant therapy or strength training paradigms) may prove most beneficial to maintain skeletal muscle mass.

**Figure 4:**
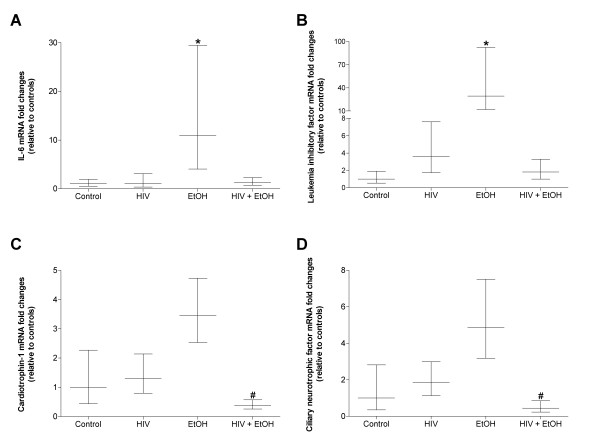
**Gene levels of IL-6 super family members are differentially regulated in diseased plantaris muscles**. As previously described [16], chronic alcohol ingestion induced expression of IL-6 (A) and LIF (B). The co-morbidity of twelve weeks of daily alcohol ingestion and HIV-1-related protein expression reduced gene expression of CT-1 (C) and CNTF (D). Data are represented as means ± range of potential values based on the 2^-ΔΔC^_T _method [15,16] and expressed as fold changes relative to controls (n = 6-7 rats/group). Significance was accepted at p ≤ 0.05. *, compared to control-fed, otherwise-healthy rats. #, compared to control-fed, HIV-1 transgenic rats.

**Figure 5:**
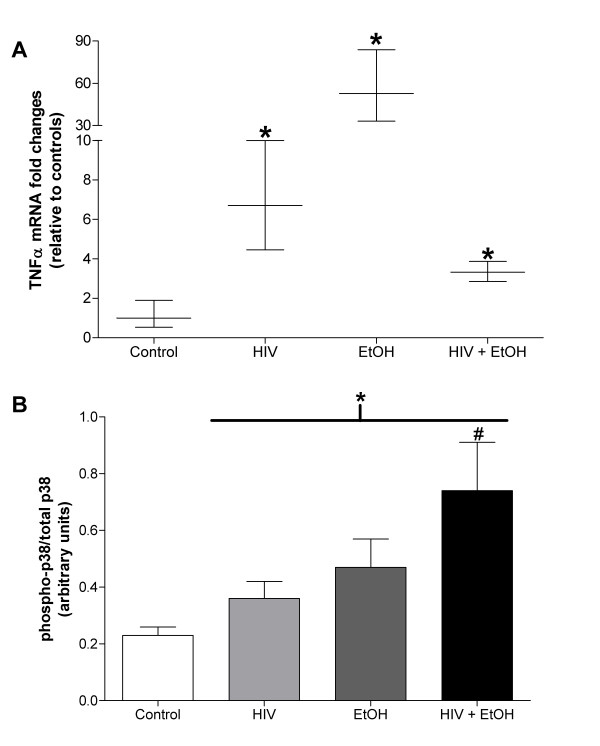
**TNFα mRNA levels and phosphorylated p38 are elevated in diseased plantaris muscles**. (A) HIV-1-related protein expression increased TNFα mRNA levels. Likewise, twelve weeks of chronic alcohol ingestion increased TNFα levels in otherwise healthy and in HIV-1 transgenic rat plantaris muscles. (B) Twelve weeks of chronic alcohol abuse increased the ratio of phospho-p38/total-p38 in each disease and co-morbid state. Chronic alcohol abuse further increased this ratio in HIV-1 transgenic rat muscle. Total p38 was unchanged in any group (data not shown). Significance was accepted at p ≤ 0.05. *, compared to control-fed, otherwise-healthy rats. #, compared to control-fed, HIV-1 transgenic rat group.

The IL-6 family of cytokines, including IL-6, LIF, CNTF, and CT-1, regulates skeletal muscle mass [16,28,29,41]. We have previously shown [16] and confirmed here (Figure 4A) that long term alcohol abuse drives gene expression of IL-6, an inflammatory cytokine associated with skeletal muscle catabolism. Similarly, twelve weeks of alcohol abuse drove expression of LIF (Figure 4B), a cytokine implicated in muscle regeneration and myoblast proliferation [30,42], and may reflect an early protective response to alcohol-induced myopathy [16]. Interestingly, this alcohol-induced expression pattern was not evident in muscles alcohol-fed, HIV-1 transgenic rats (Figures 4A and 4B) suggesting that different inflammatory cytokines may play larger roles in HIV-1 myopathies. In support of this notion, we (Figure 5A) and others [20] have reported increased TNFα mRNA expression muscle levels of in muscles from alcoholic, HIV-1 models. Together, these data strongly suggest that the myotoxic effects of chronic alcohol ingestion may manifest via multiple signaling mechanisms that ultimately depend on the underlying health status of the abuser.

The co-morbidity of chronic alcohol ingestion and HIV-1-related protein expression reduced two anabolic IL-6 family members, CNTF and CT-1 (Figures 4C and 4D), which may be partially responsible for reduced plantaris mass, protein content, and fiber area (Figure 1). In support of this notion, suppressed levels of other anabolic growth factors such as IGF-1 have been previously described in co-morbid models [10]. Interestingly, protein synthesis rates were unchanged in alcoholic, asymptomatic or SAIDS macaques [10,20] which may suggest negligible effects stemming from reduced growth factor levels. However, Molina and colleagues [10] noted that these apparent discrepancies between reduced anabolic growth factor levels and the well-established negative effects of alcohol abuse on skeletal muscle protein synthesis rates [43,44] may be due in large part to variations in alcohol feeding paradigms and circulating alcohol concentrations, or in sample collection times during the post-absorptive state.

p38 MAPK is a stress-activated protein kinase implicated in skeletal muscle catabolism [45]. Interestingly, p38 MAPK phosphorylation and subsequent pathway activation may be the result of oxidative stress or TNFα induction - two upstream factors that we and others have shown to be elevated in alcoholic and/or HIV-1 myopathies [15,16,20,46]. Here, the phospho-p38/total-p38 ratio, an index of p38 activation, was elevated in each disease state (Figure 5B) suggesting that this protein kinase plays a central role in these myopathies. Moreover, TNFα-stimulated p38 activation has been associated with downstream induction of atrogin-1 expression in *in vitro *models of muscle atrophy [33]. Atrogin-1, an E3 ubiquitin ligase, is increased in alcoholic muscle [16,27,47] or muscle from HIV-1 transgenic rats [15]. Together, these data may describe a TNFα/p38/atrogin-1 signaling pathway in alcoholic, HIV-1 myopathies.

## Conclusions

We have shown that chronic alcohol ingestion in HIV-1 transgenic rats increases skeletal muscle atrophy with concomitant losses to total protein content and relative muscle mass. These adaptations may be due in part to increased myostatin signaling, increased catabolic cytokine levels such as IL-6, TGFβ_1_, and TNFα, altered p38 pathway activity, and decreased expression of growth factors such as CNTF and CT-1. Importantly, we have previously shown that certain catabolic factors such as atrogin-1 and TGFβ_1 _as well as anabolic factors such as CNTF and CT-1 are redox-sensitive and can be modulated with glutathione restoration strategies in alcoholic muscle [16]. Interestingly, chronic alcohol feeding and a mouse model of AIDS infection (MAIDS) independently attenuated antioxidant defense capability in the liver, and in combination, led to an additive inhibition of glutathione [48]. Assuming an additive biochemical and pathological response to alcohol and HIV-1 exists in skeletal muscles, these data would strongly suggest that robust, anti-oxidant therapy with glutathione precursors such as procysteine, S-adenosyl methionine, or N-acetyl cysteine would impact an array of catabolic and anabolic factors and improve diseased skeletal muscle mass. These hypotheses are currently under investigation in our laboratory.

## Methods

### Animals and diets

Male, Fischer 344/NHsd HIV-1 transgenic rats (hemizygous NL4-3Δ *gag/pol*) [3] and wild type Fischer 344/NHsd rats (~400 g) were purchased from Harlan (Indianapolis, Indiana) and housed in pairs under a 12:12 light-dark cycle. Animals were randomized into one of four groups (n = 6-7/group): (1) wild type rats fed control diet, (2) HIV-1 transgenic rats fed control diet, (3) wild type rats fed an ethanol-containing diet, and (4) HIV-1 transgenic rats fed an ethanol-containing diet.

Rats were fed the Lieber-DeCarli liquid diet (Research Diets, New Brunswick, New Jersey) containing either alcohol or an isocaloric substitution with Maltin-Dextrin (control diet) for 12 wk [16,27]. To acclimatize the rats to the Lieber-DeCarli diet, alcohol was gradually added as 18% of total calories for 1 wk, then 27% of total calories for 1 wk, and then finally 36% of total calories for 12 wk, respectively. At the end of the 12 wk, rats were anesthetized with intraperitoneal injections of sodium pentobarbital (50 mg/kg). Muscles were removed and trimmed free of connective tissues, blotted dry, weighed, and mounted in OCT for histochemical analyses or flash frozen in liquid nitrogen for other analyses as described below. Animals were sacrificed by removal of the diaphragm muscle. All procedures were approved by Atlanta Veteran Affairs Medical Center Institutional Animal Care and Use Committee.

### Plantaris morphology & myosin heavy chain (MHC) isoform expression

Fresh plantaris muscles were embedded in OCT and immediately frozen in isopentane cooled in liquid nitrogen as previously described [15]. Serial sections from the mid-belly of the plantaris were cut at 14 or 8 μm for analyses of CSA or MHC isoform determination, respectively. All incubations were performed at room temperature. For CSA determination, plantaris sections were adhered to superfrost slides, processed for hematoxylin and eosin staining, dehydrated, and mounted. For MHC isoform determination, sections were processed for immunohistochemical detection of slow or fast MHC protein expression using the ABC method (Vector Labs, Burlingame, California). Sections were rehydrated in phosphate buffered saline (PBS, pH 7.4), incubated in blocking solution for 20 min, and then incubated in anti-slow MHC or anti-fast MHC IgG (Sigma, St. Louis, Missouri) for 90 min. Sections were washed in PBS, incubated in biotinylated secondary antibody for 60 min, washed again in PBS, and then incubated in an avidin-rich solution for 60 min. After a final wash, positive biotin-avidin binding was observed with diaminobenzidine. All sections were visualized with a Leica microscope and measured using ImageJ software (NIH, Bethesda, Maryland). Approximately 125 fibers per muscle were analyzed.

### Total muscle protein determination

Total muscle protein was determined from plantaris homogenates using previously described methods [49]. Briefly, frozen plantaris muscles were weighed, minced, and homogenized on ice using an electric tissue grinder in 40 volumes of buffer that contained: 50 mM Tris, pH 7.5, 150 mM NaCl, 0.1% SDS, 0.5% sodium deoxycholate, 1% Nonidet P-40, 1 mM PMSF, and Complete, mini protease cocktail tablets from Roche (Indianapolis, IN). Protein concentrations were determined by using the Bio-Rad DC kit according to manufacturer's instructions. Total protein per plantaris muscle was expressed as the product of protein concentration and initial wet weight. Aliquots of these plantaris homogenates were processed for protein analyses via Western blot.

### Western blot analyses

Equal amounts of protein were boiled for 2 minutes in sample buffer that contained: 0.5 M Tris-HCl (pH 6.8), 10% (v/v) glycerol, 10% (w/v) SDS, 5% (v/v) β-mercaptoethanol, and 0.05% (w/v) bromophenol blue. Samples were separated by SDS-PAGE and transferred onto nitrocellulose membranes using a trans-blot SD semi-dry transfer cell (Biorad, Hercules, CA) according to manufacturer's instructions. All incubations were performed at room temperature unless otherwise noted. Membranes were blocked in 5% BSA diluted in TTBS (0.01% (w/v) Tween-20) for 1 h and then incubated in primary antibodies against phospho-p38 and total p38 (each at 1:1000 in blocking buffer, Cell Signaling Technology, Beverly, MA) overnight at 4°C. Blots were washed in TTBS, incubated in anti-rabbit-HRP IgG (1;2500 in blocking buffer) for 1 h, washed again and then developed with enhanced chemiluminescent plus western blotting detection system (GE Healthcare, Piscataway, NJ). Densitometry was performed using a Chemidoc XRS system and analyzed with Quantity One software (Biorad, Hercules, CA). Pathway activities are expressed as the ratios of phosphorylated protein to total protein.

### Gene expression analyses

Plantaris muscles were collected, immediately frozen in liquid nitrogen, and stored at -80°C until processed for real time PCR analyses as previously described [15,16,27]. Frozen plantaris muscles were thawed and homogenized in Trizol (1 ml/100 mg tissue) using an electric tissue homogenizer. Total RNA (2.5 μg) was reverse transcribed in a 40 μl final reaction volume using random primers and M-MLV reverse transcriptase according to manufacturer's instructions (Invitrogen, Carlsbad, California).

Real time PCR products were analyzed using the iCycler iQ system (Bio-Rad, Hercules, California). cDNA (10 μl of a 1:10 dilution) was amplified in a 35 μl reaction containing 400 nm gene-specific primer pair and iQ Sybr Green Supermix (Bio-Rad, Hercules, California). Primer sequences for LIF, CNTF, CT-1, IL-6, and TGFβ_1 _have been previously described [15,16,27]. Primer sequences for myostatin, activin IIB, and TNFα were as follows: myostatin, 5'-TAACCTTCCCAGGACCAGGA-3' and 5'-GCAATAATCCAGTCCCATCC-3'; activin IIB, 5'- CGACTTTGTGGCTGTGAAGA-3' and 5'-TCGTTCCACGTGTGATGATGTT-3'; TNFα, 5'-TGGCCCAGACCCTCACACTC-3' and 5'-CTCCTGGTATGAAATGGCAAATC-3'. Samples were incubated at 95°C for 15 min, followed by 40 cycles of denaturation, annealing and extension at 95°C, 60°C, and 72°C, respectively. As a control, real time PCR was also performed on 2 μl of each RNA sample to confirm absence of contaminating genomic DNA. All reactions were performed in triplicate and the starting quantities of the genes of interest were normalized to 18S rRNA (primers supplied by Ambion, Austin, Texas). The 2^-ΔΔC^_T _method was used to analyze alterations in gene expression and values were expressed as fold changes relative to control [15,16,27].

### Statistics

One-way analyses of variance were performed followed by Student-Newman-Keuls post-hoc tests using SigmaStat v2.0 software. Significance was accepted at p ≤ 0.05.

## List of abbreviations

CNTF: ciliary neurotrophic factor; CT-1: cardiotrophin-1; CSA: cross-sectional area; IGF-1: insulin-like growth factor-1; IL-6: interleukin-6; LIF: leukemia inhibitory factor; MHC: myosin heavy chain; MuRF: muscle ring finger protein; NIAAA: National Institutes of Alcohol Abuse and Alcoholism; SAIDS: terminal stage acquired immune deficiency syndrome in SIV-infected Rhesus macaques; SIV: simian immunodeficiency virus; TGFβ_1_: transforming growth factor β_1_

## Competing interests

The authors declare that they have no competing interests.

## Authors' contributions

CC was responsible for real time PCR and histology. DG was responsible for tissue preparation, protein content analyses and data collection. MB was responsible for western blot analyses. JO was responsible for study design, research fund collection, figures and manuscript preparations. All authors have provided editorial content and have approved this final manuscript.
